# Single ultrasound-guided local high-dose thrombin injection in the treatment of giant brachial artery pseudoaneurysm: A case report

**DOI:** 10.1097/MD.0000000000030103

**Published:** 2022-08-19

**Authors:** Liang Li, Junqing Xiu, Lian Yuan, Xing Zhang, Yue Li

**Affiliations:** a Department of Ultrasound, Guang’anmen Hospital, China Academy of Traditional Chinese Medicine; b Oncology Department, South area of Guang’anmen Hospital, Academy of Traditional Chinese Medicine, China; c Surgery Department, Guang’anmen Hospital, China Academy of Traditional Chinese Medicine.

**Keywords:** brachial artery, case report, pseudoaneurysm, ultrasound-guided thrombin injection

## Abstract

**Rationale:**

Pseudoaneurysm (PSA) is a common complication related to vascular intervention, and surgical therapy is the primary method. However, a giant brachial artery PSA over 2 weeks is rarely observed. Due to the adhesion of surrounding tissue, thrombus organization, the extensive injury, and the high expense of transluminal stent-graft placement, a single ultrasound-guided local high-dose thrombin injection can be a therapy option. Such cases are rarely reported.

**Patient concerns:**

A 71-year-old man with a history of left elbow fossa interventional puncture presented to our hospital with a pulsatile mass in the left elbow fossa. He had a history of cerebral infarction 32 years prior without sequelae, emphysema for more than 2 years, hyperlipidemia for 3 months, and prostatic hyperplasia for 8 months. After conservative therapy, the lumbar compression fracture produced by trauma 24 years ago healed, and the intracranial hematoma induced by trauma ten years ago was absorbed.

**Diagnosis:**

Ultrasound examination showed giant mixed echoes on the posterior medial side of the left brachial artery.

**Interventions:**

The patient underwent a single ultrasound-guided local high-dose thrombin injection to treat giant brachial artery PSA.

**Outcomes:**

Following therapy, the ultrasonography revealed that extensive thrombosis immediately formed in the cavity, and the internal blood flow signals had completely vanished. A week later, a physical examination showed that the PSA had shrunk with no apparent tenderness and that the texture had hardened. Pulsation and vascular murmurs disappeared. Ultrasound showed that the PSA was reduced, and no blood flow signals were found.

**Lessons:**

A single ultrasound-guided local high-dose thrombin injection had a considerable effect in curing large iatrogenic PSA. However, when deciding on the best therapy, specificity must be taken into account.

## 1. Introduction

A pseudoaneurysm (PSA) is generated when the vascular wall is injured, resulting in a communicating hematoma in the surrounding tissue.^[1,2]^ Blood flows into and out of the mass when the systolic and diastolic stages shift, causing it to appear as a pulsatile mass and the blood signal to be investigated by ultrasonography. A PSA differs from a true aneurysm by the absence of all 3 vessel walls or by the waveform on duplex Doppler ultrasound.^[1,3,4]^

A PSA induced by a medical procedure is known as an iatrogenic pseudoaneurysm (IPA). PSA therapy is essential due to the possibility of rupture and bleeding. Minimally invasive ultrasound-guided thrombin injection (UGTI), ultrasound-guided compression (UGC), arterial closure devices (ACD), surgical repair, and interventional therapy are all common therapies.^[5,6]^ Clinically, the IPA of the femoral artery is prevalent. However, IPA of the brachial artery, particularly large IPA of the brachial artery, is uncommon.^[7,8]^ Furthermore, no specific clinical guidelines are currently advised.

In this case, we used ultrasound to guide a single local injection of high-dose thrombin to treat a giant brachial artery PSA. The re-examination revealed that the mass had shrunk, and there was no blood flow signal, indicating that the treatment was effective.

## 2. Case presentation

Patient consent was obtained for publishing this case report.

**Figure 1.**
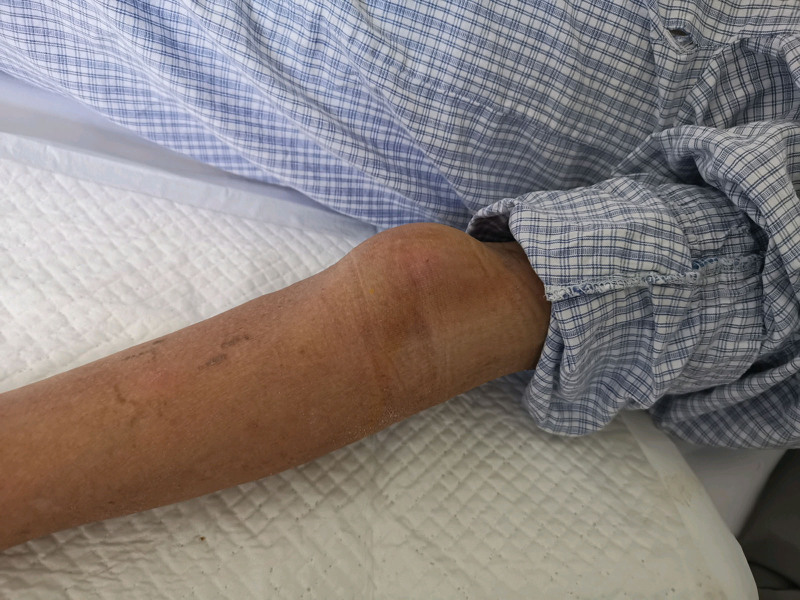
Giant psA of brachial artery caused by interventional puncture.

**Figure 2.**
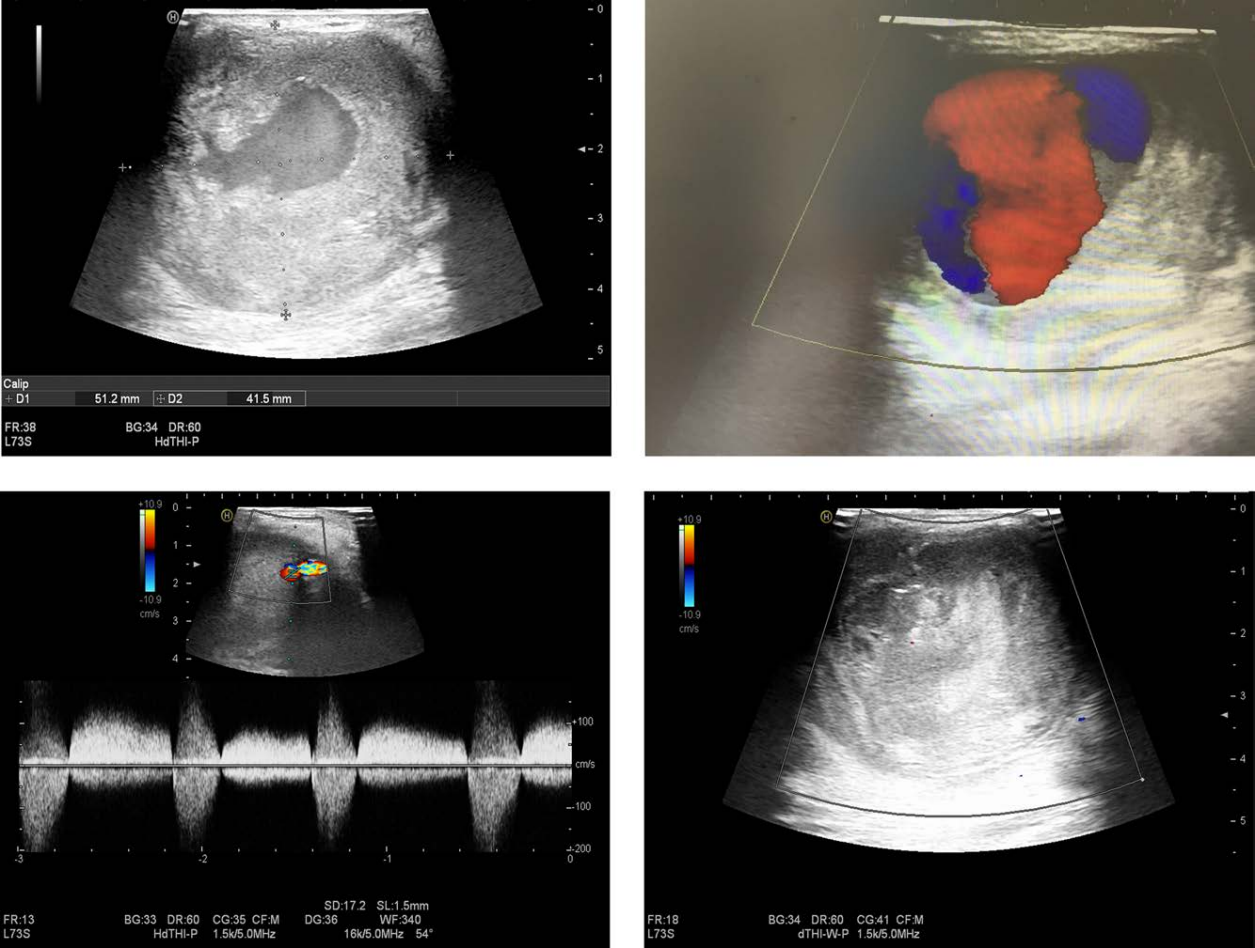
Ultrasound before and after treatment. (A) Ultrasonography showed that the mixed echo on the posterior medial side of the left brachial artery was 5.1 × 4.2 cm, accompanied by anechoic area of 3.2 × 3.2 × 3.1 cm. (B) Alternating red and blue blood flow signals can be seen, and thrombus attached on the tumor wall with 1.6 cm at the widest part. (C) Color Doppler ultrasonography showed that the anechoic area was connected with brachial artery, with an internal diameter of about 0.1cm of the psA neck, and the “to-and-fro spectrum” could be seen. (D) After injecting 600IU thrombin into the cavity of pseudoaneurysm, ultrasound showed that extensive thrombus immediately formed in the cavity, and the blood flow signals in the lumen disappeared completely.

A 71-year-old man with a history of left elbow fossa interventional puncture presented to our hospital with a pulsatile mass in the left elbow fossa. A throbbing mass measuring 10 × 12cm was observed on the medial side of the proximal end of the patient’s left elbow fossa (Fig. 1), which was tough and without tenderness, and vascular murmurs could be heard. Laboratory examination: HGB 103 g/L, CRP 10.78 mg/L, ALB 29.7 g/L, H-CRP 13.04 mg/L. Coagulation showed no obvious abnormalities. Ultrasound examination showed mixed echoes on the posterior medial side of the left brachial artery, with a size of 5.1 × 4.2 cm, accompanied by an anechoic area of 3.2 × 3.2 × 3.1 cm (Fig. 2A), alternating red and blue blood flow signals. The thrombus was attached to the PSA wall with 1.6 cm at the widest part (Fig. 2B). Color Doppler ultrasonography showed that the anechoic area was connected with the brachial artery, with an internal diameter of about 0.1cm of the PSA neck. The “to-and-fro spectrum” could be seen (Fig. 2C). The patient had a history of cerebral infarction 32 years prior without sequelae. After conservative therapy, the lumbar compression fracture produced by trauma 24 years ago healed, and the intracranial hematoma induced by trauma ten years ago was absorbed. He had a history of emphysema for more than 2 years, hyperlipidemia for 3 months, and prostatic hyperplasia for 8 months. Aspirin Enteric-coated Tablets (100 mg qd), Isosorbide Mononitrate Tablets (20 mg bid), Atorvastatin Calcium Tablets (20 mg qn), Terazosin Hydrochloride Tablets (2 mg qn), and Finasteride Tablets (5 mg qd) were among the oral medications.

Three months before, the patient had undergone an interventional puncture to treat atherosclerotic occlusive disease in the lower extremities. The treatment, however, proved unsuccessful. The patient had no clear symptoms of self-consciousness, and the mass tended to grow. Taking into account the patient’s medical history, physical examination, laboratory examination, and ultrasonic testing, we believe it was an iatrogenic large brachial artery pseudoaneurysm caused by the interventional puncture.

**Figure 3.**
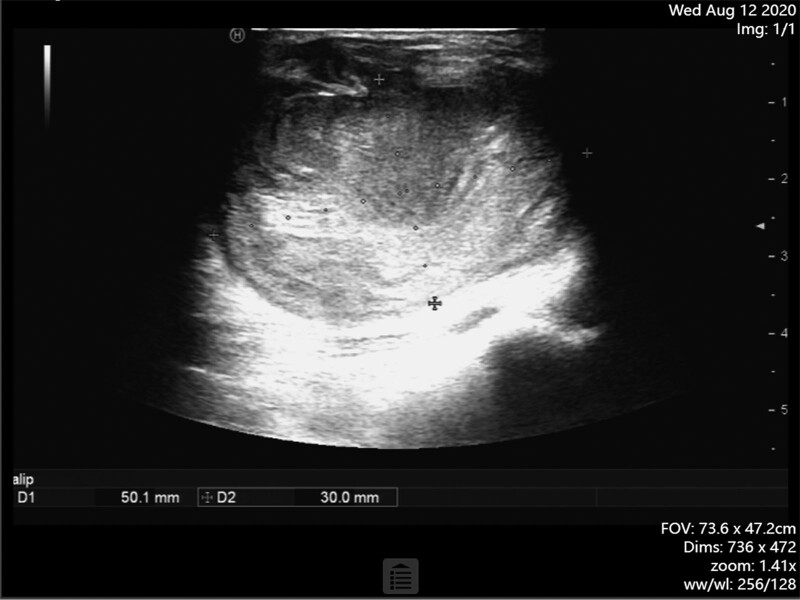
PSA had shrunk significantly, and no blood flow signal was detected a week later.

We chose a single local injection of high-dose thrombin under ultrasound guidance for this patient. After getting the patient’s informed consent, we routinely cleansed the skin, draped the area, and injected 600IU of thrombin under ultrasound guidance into the PSA cavity. Ultrasonography revealed that extensive thrombosis occurred immediately in the cavity, and the internal blood flow signals disappeared completely (Fig. 2D). A week later, a physical examination showed that PSA had shrunk, and the texture became hard. The pulsation and vascular murmur both vanished without any obvious tenderness. The patient experienced no adverse events. Color ultrasonography revealed that PSA had shrunk significantly, and no blood flow signal was detected (Fig. 3).

## 3. Discussion

With the rapid development of interventional therapy, interventional arterial puncture has been a major cause of PSA. PSA of the femoral artery is a reasonably common complication of interventional therapy, whereas PSA of the brachial artery, particularly the giant one, is rarely documented.^[9]^ With widespread use of a femoral artery puncture suture and closure device, the femoral artery PSA is drastically reduced. Generally, if a closure or suture device is used correctly, bleeding from a femoral artery puncture hole will cease instantly without any compression.^[10]^ However, there is no closure and suture device highly recommended for the brachial artery. After the puncture, compression is mostly utilized to control bleeding, and incorrect compression is the leading cause of PSA.

IPA is treated with surgical repair, interventional therapy, ultrasound-guided compression therapy (UGC), minimally invasive ultrasound-guided thrombin injection (UGTI), and arterial closure device (ACD) if conservative treatments fail.^[5,6]^ Within 2 weeks, PSA is amenable to surgical treatment, which consists mostly of arterial puncture hole repair and hematoma removal.^[11]^ Due to the thrombus organization, surgical treatment for PSA over 2 weeks is complicated. Interventional therapy, consisting of endovascular repair with a membrane-covered stent, which blocks the artery’s puncture hole from the vascular lumen, is therefore preferable in this instance.^[12,13]^ Significantly, surgical treatment is more traumatic, and interventional therapy is more expensive. In this report, the patient had a 3-month history, and the PSA was large and continuing to grow. There was no chance of self-recovery, surgical treatments were more complex, and the patient couldn’t pay the hefty cost of surgery.Clinical trials have revealed that the magnitude of PSA may impact the effectiveness of UGC.^[14]^ In actual practice, we found it difficult for compression therapy alone to be beneficial for PSA for longer than 2 weeks.

Ultrasound-guided intraluminal injection of thrombin is a great treatment for IPA due to its amazing therapeutic result, minimal trauma, rapid recovery, and inexpensive cost.^[15]^ We discovered that soon following intraluminal injection of thrombin, extensive thrombosis developed and blood flow ceased entirely. Clinical trials have shown that low-dose thrombin repeated at a specified interval can be utilized to treat small PSA. And high-dose thrombin injected at a longer time interval or low-dose thrombin injected at a shorter time interval can be employed for big PSA.^[5]^ In this case, a single injection of 600IU thrombin was applied for the giant IPA, and widespread thrombosis developed immediately. Postoperative follow-up showed no complications such as arterial thrombosis or distal embolization. Thrombin must be injected under ultrasound guidance, which allows physicians to see the dynamic connection between the PSA neck and the punctured artery. Moreover, ultrasonography can assist physicians in injecting medications into the PSA cavity rather than the arterial lumen. This gives a clinically applicable imaging approach that is real-time, safe, and effective. To prevent thrombin from entering the arterial lumen, the puncture point should be as far as feasible from the PSA neck. Otherwise, it will lead to arterial thrombosis. Generally, after effective therapy, pressure bandaging is no longer required. Theoretically, excessive pressure bandaging could squeeze the thrombus through the PSA neck and into the arterial lumen, leading to distal arterial embolization.

## 4. Conclusion

Our findings indicated that a single ultrasound-guided local injection of high-dose thrombin had a considerable impact on iatrogenic giant brachial artery PSA. During folow-up, no postoperative complications such as arterial thrombosis were observed. For the therapy of giant PSA, the individual circumstances must be considered. To investigate the safety and efficacy of this method, future research with bigger sample numbers should be done.

## Author contributions

Conception and design: L.L. and L.Y.; writing the article: L.L. and L.Y.; critical revision of the article: X.Z. and J.Q.X.; Finally, all authors read and approved the final version of the manuscript. Overall responsibility: L.Y.
